# When I say … clinically significant change

**DOI:** 10.3389/fresc.2026.1913834

**Published:** 2026-08-11

**Authors:** Christoph Lindner, Steffen Zitzmann

**Affiliations:** 1 Department Clinical Translation & Research Hospital, Max Planck Institute of Psychiatry, Munich, Germany; 2 Department of Psychology, Medical School Hamburg, Hamburg, Germany

**Keywords:** clinical decision-making confidence, clinically significant change (CSC), human functioning sciences, medical education, recovery, treatment response

## Introduction

Imagine a patient sitting in front of you after eight weeks of inpatient treatment or rehabilitation. At admission, her clinical condition was severe, and a blood marker, imaging finding, or expert rating still indicated active disease; today, these indicators look better. She sleeps better, leaves the ward more often, and says that everyday activities feel less exhausting. The team now has to decide whether to continue inpatient treatment or rehabilitation, step down to outpatient care, or change the treatment plan. Someone asks: has this patient's condition changed in a clinically significant way?

When I say clinically significant change, I mean the interpretation that an individual patient's condition has changed enough to matter for clinical understanding, communication, or care. The question is not merely whether an indicator of the patient's condition, such as a clinical observation, biomarker, imaging finding, or test score, has changed, but whether this change is meaningful for clinical decision-making and patient care. This distinction is central to rehabilitation and the human functioning sciences, where health is understood not only in terms of biological processes and disease indicators, but also in terms of what people are able to do in everyday life and in their environments (1). Drawing on established approaches to recovery, response, and additional reference points, this opinion article provides clinicians with an accessible, evidence-informed orientation to interpreting clinically significant change in individual patients. It explains how established approaches can inform clinical communication and decision-making.

## Recovery, response, and additional reference points

One way of defining clinically significant change is to focus on the patient's status after treatment. A post-treatment score that crosses a predefined clinical threshold, such as falling below a symptom cut-off score, may support the notion that the patient is no longer within the clinical range. In simple words, this addresses the recovery question of whether the patient's current state still indicates clinical illness, or whether she is now in a more functional state. Jacobson and Truax framed this as movement from a dysfunctional to a functional range, while also requiring that the observed change be reliable in order to support a clinical interpretation (2, 3).

Recovery, however, is not the only clinically meaningful outcome. A patient can improve substantially but still remain clinically ill. A person with a severe inflammatory, oncological, neurological, or psychiatric condition may show a substantial decrease in a blood biomarker, a reduction in lesion burden on imaging, or a large improvement on a clinical rating scale. The person still has symptoms, but the improvement is large and may thus be highly meaningful: the patient may get out of bed again, speak with family, eat regularly, and no longer feel trapped in hopelessness. Calling this “not clinically significant” may be technically correct from the status-oriented definition but clinically incomplete.

Therefore, a second definition asks whether the patient has improved enough compared with where she or he started (4–7). This perspective is particularly useful when full recovery is not a realistic clinical aim, or when the aim is meaningful relief rather than recovery. In this sense, clinically significant change addresses the question whether the patient has responded. The patient may still be ill, but symptoms, functioning, risk, or need for care have changed enough to matter.

A third, broader definition links the measured change to clinically meaningful additional reference points beyond the test used (8). Such additional reference points may be used, for example, to derive a minimal important difference (MID), that is, the smallest change in an outcome score considered important by patients or clinicians, or a minimal clinically important difference (MCID), that is, the smallest change perceived as beneficial and potentially warranting a change in treatment (6, 7). Jaeschke and colleagues (6) illustrated this MCID approach by relating changes in test scores to patients’ reports of change, whereas Revicki and colleagues (7) recommended using multiple reference points based on patient reports and clinical indicators to estimate the MID. More broadly, such reference points may also be considered jointly to support the interpretation of an individual patient's change without being reduced to a single numerical threshold (7, 8). The patient may report whether she notices meaningful differences in daily life. Clinicians, nurses, relatives, or caregivers may judge whether the patient is more engaged, participates more in ward routines, needs less support, or whether the current state justifies a change in treatment. Such additional perspectives do not replace clinical indicators such as clinician-rated scores, biomarkers, or imaging findings, but help determine whether the observed improvement is meaningful for the patient's everyday functioning. Their interpretation should be guided by explicit criteria and, where possible, structured assessment procedures.

## Discussion

Table 1 organizes the three definitions of clinically significant change: recovery, response, and additional reference points. The table provides guidance on how to choose among these definitions based on the clinical question. Has the patient recovered? Has treatment helped? Does the test provide sufficient information, or should patient experience, ward observations, relatives’ observations, functioning, and likely future course be considered as well? We note that the definitions may also be applied in combination. For example, a blood marker or imaging finding may improve, while the patient still mobilizes little and needs substantial support. Such disagreement is not a nuisance but may indicate that biological response and functional recovery do not yet point in the same direction.

**Table 1:** Three practical questions that may guide the interpretation of clinically significant change.

Clinical interest	Possible question	May be useful when	Possible interpretation
Recovery	Is the patient's current state now outside the clinical range?	The decision concerns remission, discharge readiness, or return to a more functional state.	Recovered/not recovered
Response	Has the patient improved enough compared with where she or he started?	Full recovery is unlikely or not the main aim, but meaningful relief matters.	Improved/unchanged/deteriorated
Additional reference points	Do the patient's experience, structured clinical assessment, nursing observations, relatives’ perspectives, functioning, and follow-up course support the same interpretation?	Test scores, imaging findings, or other clinical indicators are difficult to interpret, or the decision is clinically consequential.	Supported clinical interpretation

The table is an author-developed synthesis informed by the literature on recovery and remission (2, 3, 5), treatment response (4, 5), and the use of clinically meaningful additional reference points (6–8).

Across all three approaches, the quality of the interpretation depends not only on the clinical relevance of the chosen reference point but also on the objectivity of the assessment process. When assessment involves subjective judgments to a large degree, they should be considered alongside information from standardized clinical interviews, validated questionnaires (i.e., self-reports), and, where appropriate, structured information from relatives, caregivers, or other informed observers (7, 8), or an assessment by a clinician not directly involved in treatment in order to reduce observer-related bias (9). In conclusion, we presented definitions of clinically significant change that are most prominent in the field, while acknowledging that they answer somewhat different clinical questions. When I say clinically significant change, I mean a clinically defensible interpretation of individual patient change after treatment. The central question is whether the patient improved in a way that matters.

## References

[1] Bickenbach J Boggs D Prodinger B Stucki G. Establishing the human functioning sciences sections in frontiers in rehabilitation sciences. Front Rehabil Sci. (2026) 7:7. 10.3389/fresc.2026.1768887 PMC13102811 42040648

[2] Jacobson NS Truax P. Clinical significance: a statistical approach to defining meaningful change in psychotherapy research. J Consult Clin Psychol. (1991) 59(1):12–9. 10.1037/0022-006X.59.1.12 2002127

[3] Lindner C Roell L Falkai P Zitzmann S. Beyond the reliable change index – misunderstandings and myths surrounding the Jacobson–Truax method. Psychol Test Adapt Dev. (2026) 7:296–302. 10.1027/2698-1866/a000144

[4] Zitzmann S Lindner C. How to assess response. Eur Arch Psychiatry Clin Neurosci. (2025) 275(5):1531–2. 10.1007/s00406-024-01834-8 38839710 PMC12270965

[5] Leucht S Davis JM Engel RR Kissling W Kane JM. Definitions of response and remission in schizophrenia: recommendations for their use and their presentation. Acta Psychiatr Scand. (2009) 119(s438):7–14. 10.1111/j.1600-0447.2008.01308.x 19132961

[6] Jaeschke R Singer J Guyatt GH. Measurement of health status. Ascertaining the minimal clinically important difference. Control Clin Trials. (1989) 10(4):407–15. 10.1016/0197-2456(89)90005-6 2691207

[7] Revicki D Hays RD Cella D Sloan J. Recommended methods for determining responsiveness and minimally important differences for patient-reported outcomes. J Clin Epidemiol. (2008) 61(2):102–9. 10.1016/j.jclinepi.2007.03.012 18177782

[8] Crosby RD Kolotkin RL Williams GR. Defining clinically meaningful change in health-related quality of life. J Clin Epidemiol. (2003) 56(5):395–407. 10.1016/S0895-4356(03)00044-1 12812812

[9] Hróbjartsson A Thomsen ASS Emanuelsson F Tendal B Hilden J Boutron I. Observer bias in randomized clinical trials with measurement scale outcomes: a systematic review of trials with both blinded and nonblinded assessors. Can Med Assoc J. (2013) 185(4):E201–11. 10.1503/cmaj.120744 23359047 PMC3589328

